# Hypertensive patients’ knowledge and practices on lifestyle modification in Extension 6, Middelburg

**DOI:** 10.4102/safp.v64i1.5528

**Published:** 2022-08-24

**Authors:** Amir Rahimi, Lushiku Nkombua

**Affiliations:** 1 Department of Family Medicine, Faculty of Health Sciences, University of Pretoria, Pretoria, South Africa

**Keywords:** hypertension, knowledge, practice, beliefs, lifestyle modification, control

## Abstract

**Background:**

Hypertension (HTN) is one of the most common chronic diseases affecting the majority of patients worldwide, including in South Africa. The control of HTN and prevention of complications are major challenges for patients and healthcare workers. The proper control of the disease requires a multifactorial approach consisting of medical treatment, as well as lifestyle modification, with the assistance of healthcare workers. Addressing patients’ beliefs, the practice of lifestyle and acceptance of change are some of the ways of aiding control.

**Methods:**

The researchers used a cross-sectional and descriptive survey to establish the knowledge and practices of lifestyle modifications in patients with hypertension. A validated questionnaire was adopted. A total of 250 participants from the Extension 6 Clinic in Middelburg, Mpumalanga, constituted the study population.

**Results:**

Most of the participants had borderline high blood pressure (37.2%) or uncontrolled high blood pressure (46%). The participants’ knowledge of HTN and its complications was not adequate. The study established that increased age and long duration of HTN were associated with high numbers of uncontrolled HTN. Most of the participants (88.8%) had difficulty exercising. Also, most of the participants (90.8%) did not have a place or facility for exercises. Fifty per cent of the participants were unable to maintain a balanced healthy diet which included fruits and vegetables.

**Conclusion:**

The study explored the participants’ knowledge and practice of lifestyle modification. The participants lacked knowledge regarding the definition of HTN and the meaning of controlled HTN. To assist the patients and improve on the identified pitfalls, each consultation session should include some methods of education, and motivation for healthy behaviours and lifestyle modification. This should be extended to all the people visiting the health facilities for them to adopt a healthier diet, greater intake of vegetables and availability of fitness facilities for the community.

## Introduction

Hypertension (HTN) is one of the major health problems worldwide, affecting a high percentage of the adult population. Fast urbanisation and changes in peoples’ lifestyles, such as poor diet, lack of physical exercise, increased alcohol intake, and smoking, contribute to the increase in the incidence of HTN.^1^ The growing number of people with HTN are at risk of suffering from cardiovascular and renal complications if the disease is not well controlled.^2,3^ It is important to note that patients need to be made aware of such complications for better prevention. Reports have indicated that poor control of HTN is related to the lack of knowledge of patients about HTN and healthy lifestyle modification.^4^ Control of HTN and its associated complications is considered a health challenge globally, especially in developing countries, such as South Africa, which are spending large amounts of their annual budget on the problem.^5^ Policymakers and health practitioners tried to improve these challenges with more cost-effective ways, such as non-pharmacological healthy lifestyle methods.^6^ The national guidelines of HTN of South Africa highlighted the importance of lifestyle modification in the control of HTN, namely, ‘lifestyle counselling is the cornerstone of management’.^1^ Patients’ beliefs, together with their level of knowledge about lifestyle modification, directly influence the control of HTN and the prevention of complications, such as cardiovascular disease and renal disease.^2,3^ Complications of HTN, such as strokes, cardiovascular diseases and renal disorders, are life-threatening and cost-consuming. Pharmacological management of HTN is costly and could have associated side effects depending on the drugs being used. Adopting a healthy lifestyle either in combination with medication or as a sole alternative for preventing HTN is an effective contributor to controlling HTN and its complications.^7^ The researchers wanted to assess the level of knowledge of hypertensive patients regarding different aspects of lifestyle modification for the control of high blood pressure (BP). The results of the study may help policymakers plan for the health education of the community as well as assist healthcare providers to include non-drug modalities in the management of HTN. Further basic information can also be a milestone for further research in the area.

## Methodology

This was a cross-sectional and descriptive survey. The research site was the primary health clinic (PHC) in Extension 6, Mhluzi Township, Middelburg, Mpumalanga.

Mpumalanga (Zulu name for ‘the place where the sun rises’) is a province in eastern South Africa with an area of 76 465 km^2^, the eighth biggest area ranked among the nine provinces with a population of 4 036 939, which is the sixth largest in terms of population in South Africa. Mpumalanga province is divided into three districts: Nkangala, Ehlanzeni and Gert Sibande. Middelburg is in the Nkangala district and the smallest area among the three districts with an area of 16 755 km^2^ and the second largest in terms of population, which is 1 445 624, with the highest population density of 86.3 per km^2^ in the province.^8^

The study population consisted of adult patients with HTN attending Extension 6 Clinic, Middelburg, Mpumalanga, during the autumn of 2019.

### Inclusion criteria

In order to be included in the study, the participants had to be 18 years old or above, mentally competent and able to give informed consent.

### Exclusion criteria

Patients who could not understand the questions and those who declined to consent were not included.

### Data collection

Data were collected from using a directly administered questionnaire. The questionnaire was peer-reviewed, developed and used by Weir et al.^9^ and Gazmararian et al.^10^ This questionnaire was modified to fit the context of the researcher’s practice. The questionnaire focused on the knowledge and practice of lifestyle modifications for the management of HTN. The knowledge questions are designed to measure the participant’s concept regarding issues that are communicated to patients with HTN. Demographic data (age, gender, duration of illness and education) were also collected. The data were captured without divulging the participants’ names. Blood pressure was measured by means of an electronic BP machine (Dynamap), while the participants were in a sitting position with their back resting against an upright object, and their arms positioned at heart level. The participant rested for at least 30 min before BP was measured. The first measurement was discarded, and thereafter measurements were done three times at 5-min intervals. The highest recording was then recorded. Weight was measured with a standard balance scale available in the clinic. Body mass index (BMI) was calculated by dividing the weight (kg) by height (m^2^). In this study, HTN was classified according to the Southern African Hypertension Society (2014).^1^ The three categories, namely, normal, optimal and high normal, were considered controlled HTN, while grade 1 was considered borderline, and grades 2 and 3 were considered uncontrolled HTN in this study.

### Statistical considerations

In this study, the knowledge and practice towards HTN control by means of lifestyle modifications were assessed. The sample size of this study was based on the estimation of the expected proportion of 0.25 of patients that will have adequate knowledge. A sample of at least 240 participants was estimated, with 95% confidence interval (CI) that the expected proportion to accuracy is within the 0.05 level of significance. This sample size was found to be adequate to assess the association between demographic data and clinical variables with patients’ knowledge. No more than 12 variables were expected to enter the multiple logistic regression, and with an expected proportion of 0.25 a sample of at least 200 patients was required.^11^

### Data analysis

Data collected from the questionnaires were summarised using description statistics that made use of frequency tabulation, continuous variable summaries using, but not restricted to, the number of descriptive statistics, mean, standard deviation, median, range, interquartile range, and 95% CI. Categorical variables summarised using frequency, proportion (percentage), cross-tabulation, and 95% CI. Modelling the outcome’s adequate knowledge, adequate practice, and good control against demographic and clinical variables made use of multiple logistic regression. The statistics of interest included odds ratio, along with a 95% CI testing at the 0.05 level of significance.

### Ethical considerations

Approval was obtained from the Family Medicine Department Research Ethics Committee and the University of Pretoria, reference number: 427/2017, and authorisation was obtained by the Health Facility Management in Middelburg Hospital. Informed consent was obtained from all participants. The data were captured using participants’ numbers, not by their names to ensure confidentiality.

## Results

A total of 250 participants were recruited for the study. The results are presented in three sections, namely, demographics/anthropometrics, healthy lifestyle practices, and the knowledge of HTN by the participants.

### Demographic and anthropometrics

Physical characteristics and anthropometrics for two of the participants (weight, height, and BMI data) were not possible because they were wheelchair bound (Table 1).

**TABLE 1:** Physical characteristics.

Variable	Observations	Mean	s.d.
Age	250	50.37	12.25
Height	248	1.59	0.07
Weight	248	78.25	14.65
BMI	248	30.91	5.87
Waist	250	85.74	17.25
SBP	250	137.26	17.62
DBP	250	84.53	11.70

s.d., standard deviation; BMI, body mass index; SBP, systolic blood pressure; DPB, diastolic blood pressure.

#### Gender distribution

This included 108 men (43.20%) and 142 women (56.80%).

#### Age, height, weight, waist circumference and BMI

The participants’ ages ranged between 20 and 86 years. The average age for men was 49 years and for women 51 years (Table 2).

**TABLE 2:** Age, height, weight, waist circumference and body mass index distribution based on gender.

Gender	Frequency	Cumulative frequency	Mean age	Mean height	Mean weight	Mean waist circumference	BMI
Male	107	107	49.673	1.608	75.928	81.673	29.428
Female	143	250	50.888	1.581	79.976	88.783	32.016

**Total**	**250**	**-**	**-**	**-**	**-**	**-**	**-**

BMI, body mass index.

Comment: Mean weight was more than 80 kg and mean BMI was more than 32 for women and, 29.428 for men. The waist circumference average for women was 89 cm compared to 82 cm for men.

#### Educational status

Among the participants, 51 (20.40%) did not attend any school, 67 (26.80%) completed primary schooling, 105 (42.00%) attended high school and only 28 (10.80%) had a tertiary education qualification.

#### Employment status

Most of the participants were unemployed, 136 (54.40%), or temporarily employed as labourers, 47 (18.80%). There were 39 semiskilled workers (15.60%) and 28 professional workers (11.20%).

#### Blood pressure control in the participants

The mean systolic BPs were higher in the male participants (138.9) compared to the female participants, who had a mean systolic BP of (136.0). Mean diastolic BPs were also higher in male participants (86) compared to that in female participants (83.4).

### Hypertension knowledge and beliefs among participants

#### Participants’ definition of hypertension

Most of the participants (87.70%) responded that they knew what HTN was compared to 12.30% who did not know. Six participants did not answer this question.

#### Duration of hypertension in the participants

Most of the participants, 133 (55.19%), had HTN for less than five years, 558 (24.07%) between 5 and 10 years, 33 (13.69%) were diagnosed less than one year ago and 17 (7.05%) more than 10 years ago. Nine participants were uncertain about the date of the diagnosis.

#### Participants’ source of information

Most of the participants, 172 (73.19%), obtained information regarding HTN from the nursing personnel, and 53 (22.55%) from physicians, 1 (0.43%) from reading material, and 9 (3.38%) from other sources of information.

#### Participants’ ideas about their blood pressure control

The majority of the participants (85.2%) believed that their BPs were controlled, with more women thinking that way than the men, which contradicts the measured BP that revealed uncontrolled HTN in the majority of participants.

#### Participants’ knowledge about hypertension syndrome (warning signs)

A total of 39.6% of participants knew about the warning signs of high BP, 54.8% did not know about the warning signs, and a few did not answer this question (5.6%).

#### Participants’ knowledge about complications of hypertension

The majority of the participants (78.0%) knew that stroke was a complication of HTN, 10.4% knew of heart attack, 4.4% mentioned kidney disease, and 0.8% the hardening of arteries. A few (6.4%) did not know any complication of HTN.

#### Effect of diet and salt on hypertension

The majority of the participants’ (90.8%) opinions were in agreement with the importance of diet in HTN, and 97.6% agreed that salt reduction aids HTN control.

#### Effect of regular exercise

The majority (86.8%) agreed that maintenance of regular exercise helps in control of high BP.

#### Effect of cigarette smoking on hypertension

The majority of the participants (52.8%) did not agree with this statement that cigarette smoking is not bad for HTN if less than 10 sticks are smoked per day.

#### Alcohol intake is good for hypertension

More than 50% of the participants did not agree, 10% agreed and added that limited amount of alcohol consumption was good, while 12% agreed and commented that the consumption of alcohol is good for high blood pressure (HBP) and 18% did not know about the effect of alcohol on HTN.

### Lifestyle practices of the participants

This section explores the monitoring of BP and daily practices of the participants:

- **Frequency of BP check-up:** More than 60% of the participants had a BP check-up at least once a month. The rest of the participants had their BP checked at intervals of more than a month.
- **Persons measuring the blood pressure:** Of the participants, 31.2% of measurements were done by a physician, 66.4% of the BP measurements were done by the nurses and only 2.4% of the participants’ BP measurements were done by themselves (self-measurement).

### Measures taken by participants to control blood pressure and challenges encountered

Most of the participants (79.2%) used both prescribed medications and lifestyle modification to control their BP. They also mentioned that they were careful when making choices about the food they consumed in order to control their weight. However, many of them (84.4%) alluded to the fact that it was not easy to stick to the recommended diet. Most of them (87.0%) also knew that avoidance of fatty food was beneficial for HTN. Furthermore, an important percentage of the participants (90.4%) avoided foodstuff with a high salt content. Regarding consumption of fruits and fresh vegetables, only 51.0% of the participants mentioned that they consumed them at least once a day. When participants were asked about their body weight, many of them (78.8%) were satisfied with it. As far as exercises were concerned, 80.0% of the participants denied doing any, while 8% considered walking and running as their way of exercising. The majority of the participants mentioned some limitations as factors for them to exercise, such as hard-to-follow recommended exercises (88.8%) and lack of suitable place/area for exercises (90.8%). Regarding enquiries about smoking and alcohol consumption, only 18.0% of the participants (predominantly men) were smokers and only 22% consumed alcohol beverages.

The authors also enquired if there were stressors in the participants’ lives, and the majority (84%) denied having stressors at the time of the study.

## Discussion

The objectives of this study were to determine what proportion of the hypertensive patients were well controlled through the assessment of their knowledge regarding lifestyle modifications and their practice of the said lifestyle modifications. The majority of them were found to be not controlled and had poor knowledge and practices of lifestyle modifications. The details are discussed below.

Most of the participants were overweight and had a high BMI (mean BMI for men 29.4 and women 32.0). According to the SAHS criteria, the ideal BMI range should be between ‘18.5 and 24.9’.^1^ Serafim et al.^12^ found that, in uncontrolled hypertensive patients, the BMI was at the upper limit of overweight (29.04 ± 4.35 kg/m^2^). Feng et al.^13^ also established that there was a relationship between obesity and chronic diseases such as HTN; BMI of more than 24 kg/m^2^ in both sexes was strongly associated with HTN. Other authors also confirmed the correlation between high BMI, high waist circumferences and HTN, which all lead to cardiovascular complications.^14,15,16^ Our study population may have also been at risk of cardiovascular complications.

### Educational status

The majority of the participants in the study did not attain the level of high school. It has been established that education empowers people to take care of their health issues. Rizvi et al.^17^ reported that good level of education and physical activity had a significant effect on reducing and controlling HTN. Veghari et al.^18^ also described a relationship between the level of literacy of patients and control of HTN. Powers^19^ and Yilmazel^20^ had also reported this relationship between the level of literacy of patients and prevention, diagnosis, and control of HTN. The findings in this study concurred with what had been reported elsewhere. The findings may account for the low level of controlled HTN in the participants (< 20% for systolic blood pressures [SBPs] and < 30% for diastolic blood pressure [DBP]).

### Employment status

More than 54% of the participants were unemployed, 19% had temporary jobs as labourers, and only 27% had steady jobs. Unemployment could have been a reason for the low level of practice of lifestyle modification because those participants might have had financial difficulties. These findings concur with those reported by Ntuli et al.^21^ in Limpopo province. Similarly, Nwosu^22^ established a strong relationship between unemployment and the high prevalence of all types of chronic illnesses, including HTN. In the same vein, Nygren et al.^23^ reported that there was a significant relationship between unemployment in the youth and HTN in older age groups.

### Blood pressure control in the participants

The study established that many of the participants had uncontrolled HTN. The average high age of the participants as well as their low level of education could have been a contributing factor to the uncontrolled HTN. Lower levels of knowledge of healthy lifestyle and low levels of practice of lifestyle modification also had an effect on the high percentage of uncontrolled HTN as highlighted in the SAHS guidelines.^1^

### Knowledge and beliefs of the participants regarding hypertension

Most of the participants volunteered that they knew what HTN was. However, when they were asked about the symptoms/signs of HTN and its complications, many were found not knowledgeable. The definition of HTN widely differed in the patients’ perspectives as reported in the literature.^5^ These findings correlated with the results of our study. Most of the participants stated that they had received education regarding HTN from the nurses, and a few of them from the physicians (doctors). In Peduzzi et al.,^11^ it was well established that using educational materials increased the chance to improve the level of knowledge, and practice of lifestyle modifications.

Many of the participants believed that their HTN was controlled. This may have been a sense of false assurance because only a few of them had normal BP readings. Similar findings have been reported by Lotika et al.,^24^ where the majority of their study’s subjects did not have correct information about HTN and its control. It was evident that most of the participants did not have enough knowledge and information regarding the warning signs.^5^

### Duration of hypertension

It may be assumed that the longer an individual had a disease the more knowledge they may have had about the disease and its complications. Tsioufis et al.^25^ discussed the resistance to HTN management as a factor related to the duration of the diagnosis of HTN. Our study found that the shorter the duration of diagnosis of HTN, the lower the control rate of HTN. On the contrary, Raskeliene et al.^26^ reported that the duration of disease in patients with good control of HTN is no different with non-hypertensive patients compared to the patient with poor control of HTN.

### Diet, reducing salt intake and regular exercise in treatment and control of HBP

The results of the study showed that the participants’ awareness was good about the importance of diet and reducing salt in the control of HTN. Jessen et al.^27^ reported similar results, where the majority of their subjects had awareness about reducing salt intake. Some authors revealed some complacency in their patients; Alawwa et al.^28^ and Wicaksana et al.^29^ showed that even with good awareness of reducing salt intake there was still a high rate of uncontrolled HTN because of the low rate of practising this behaviour by patients.^28,29^ This concurs with the findings of our study because of the uncontrolled BP in most of them. Dietary actions to stop HTN not only included salt reduction but also other measures, such as consumption of fruits, vegetables, grains and legumes.

Exercise: The results of the study showed that the level of participants’ knowledge was good as they reported that regular exercises reduced the BP. This has been established by Meilhac et al.^30^ and Somani et al.^31^ on the benefits of exercises in the control of HTN. Awotidebe et al.^32^ determined that the low percentage of knowledge about exercises in a Nigerian population resulted in a high rate of uncontrolled BP in hypertensive patients.

### Alcohol intake and cigarette smoking in the participants

The majority of the participants in this study were neither drinkers nor smokers. They seemed not to understand the impact of these habits on HTN.

### Measures taken by participants to control blood pressure and challenges encountered

The majority of the participants had their BP checked monthly. This may have indicated that most of the participants checked their BP regularly. It may have also implied good attendance and follow-up of patients in the clinic. Guthmann et al.^33^ found that there was a direct relationship between the frequency of return visits for BP checks and the control of HTN. Their findings correlated with the results of our study regarding the need for more frequent check-ups and follow-ups of patients in the clinic. Thus, in the majority of participants BP was measured by the nursing personnel during the monthly visit. Therefore, if the teaching of BP measurement was done properly, it did not matter if it is checked by a nurse or a doctor.^1^ Beevers et al.^34^ emphasised on the correct methods of measuring BP. The latter study showed that if correct methods of BP measurement were taught and practised by different levels of primary healthcare providers this would have had a positive impact in controlling HTN.

Most of the participants used both prescribed medications and lifestyle modification to control their BP. This finding established that there was a high level of awareness in patients about lifestyle modification, but they may not have properly practised them. These findings were supported by the reports from Jolles et al.^5^ regarding patients’ perspective, and Magadza et al.^3^ regarding patients’ beliefs and knowledge about HTN. Concerning lifestyle modification, Collier et al.^35^ found that lifestyle modification was beneficial as an HTN preventative measure. It could have been continued through diagnosis to control HTN.

### Recommended diet and weight control

The majority of the participants said that they controlled their weight by being careful about what they ate. This finding contrasted with the existence of a high percentage of increased BMIs and waist circumferences in the participants. It showed that despite the perception of most participants regarding good awareness about healthy diet, this did not translate into a healthy body weight. This had also been found in other studies by Feng et al.^13^ regarding BMI and HTN, and Song et al.^14^ regarding HTN in patients who were overweight. Undoubtedly, most of them reported that it was difficult to follow the recommended diet regimens. This has been confirmed by other authors, such as Zhang et al.,^15^ regarding obesity-related HTN, and Ostchega et al.,^16^ regarding abdominal obesity and HTN. These results showed that despite the perception of good awareness about healthy diet to control HBP, it was difficult for the patients to practice it. Therefore, dietary advice was important in the management of HTN. Goncalves Torres et al.^36^ established that a dietary counselling program could lead to the long-term effect of weight loss and better control of HBP in hypertensive patients.

Among the dietary actions, avoidance of fatty food and foods with a high sodium content were practised by the majority of the participants as evidenced by their answers to the specific questions.

The South African HTN Guidelines by Seedat et al.^1^ also emphasised the importance of salt intake restriction in the control of HTN. Fruit intake and fresh vegetables are also important in the control of HTN, and only half of the study population mentioned that they consumed these on a daily basis. The other half was not aware that it was necessary to consume these.

This was shown by Jolles et al.^5^ regarding patients’ perspective on HTN and Borgi et al.^37^ regarding the benefits of consuming fruits and vegetables. Borgi et al.^37^ showed that the benefits of the daily use of vegetables in the control of HBP, proper education and follow-up motivations could improve this kind of healthy practice in hypertensive patients.

Although most of the participants were overweight as shown by their BMIs, they still had the impression that their body weight at the time of the study was normal. Okop et al.^38^ also reported that although all the participants in their study agreed on the dangers of being overweight in uncontrolled HTN and its complications, their perception of being overweight was underestimated, especially among the female population. The latter concurred with our findings.

### Exercise frequency and types

Exercise has a positive effect on the prevention, treatment and control of HTN, as reported by Pescatello et al.^39^ The vast majority of study population found it difficult to exercise. They added that they did not have a place for exercising. The findings may have interpreted that the main reason for the low percentage of participants exercising was the unavailability of exercise facilities in the community. Furthermore, very few said that they understood that walking and running were the only forms of exercises. This was corroborated by Somani,^31^ Comelissen et al.,^40^ and Zeigler et al.^41^ It is common knowledge that walking and jogging in the streets and walkways are good types of exercises; however, security threats are deterrents for many people in South Africa to undertake them. Lastly, the participants were assessed about the level of stress in their lives as it has been reported that stress may negatively impact BP control. Most of them did not have stress nor was stress a dominant factor in their daily livelihoods. Although Kang^42^ established a relationship between stress and moderately high uncontrolled HTN, Agyei et al.^43^ found that there was no significant relationship between stress and control of HTN.

## Recommendations

Hypertension management has shown the importance of lifestyle modifications. Lack of the participants’ knowledge regarding the definition of controlled HTN and the absence of practice of good lifestyle are important challenges in the healthcare system. The need for some interventions with use of ‘quality improvement projects’ and community-oriented primary care (support groups) is essential. Conveying knowledge to patients who are illiterate will require more research. More time must be spent on the accomplishment of the educational demands in the healthcare system. This may guide patients with a lack of knowledge regarding their disease (HTN) and management (medication and lifestyle modification) thereof.

According to one of the principles of family medicine in primary healthcare, all healthcare practitioners must use every encounter as an opportunity for health education and promotion of health. Healthy lifestyle modifications should be included in the curriculum of health sciences education. This should be done by implementing national public health strategies and policies, such as limitation of use of salt in the commercial products like bread, and reducing the use of high-cholesterol products, such as animal fat and even unsaturated cooking oil.

### Strengths and limitations

The study was done in a primary care setting (PHC clinic), and as such it is very relevant to the field of family medicine. The study had some limitations: it was a cross-sectional and descriptive survey on a directly administered questionnaire, with self-reported answers. Therefore, the findings may not have been generalised.

## Conclusion

Hypertension is one of the major health problems throughout the world. It is one of the biggest causes of death throughout the world and is responsible for challenging complications, such as cardiovascular and cerebrovascular complications, consequently resulting in high morbidity and mortality rates. These challenges can be addressed by multipronged approaches based on close observation, adherence to medications and lifestyle modifications. The objectives of this study were to determine what proportion of our hypertensive patients were well controlled through the assessment of their knowledge regarding lifestyle modifications and their practice of the said lifestyle modifications. The study established that a very low percentage of participants had controlled HTN. Multiple factors may explain the low percentage of controlled BP in this study’s population. One of the factors is the presence of a high BMI resulting in participants who are overweight. This is also because of the profound lack of sufficient knowledge regarding lifestyle modification. Absence of a healthy diet and quasi-inexistence of regular physical exercises were found in our study. The study also established that participants had additional problems compounding the control of HTN, such as the lack of adequate knowledge of HTN and its complications, high unemployment and poor financial stability.
